# Picornavirus translation strategies

**DOI:** 10.1002/2211-5463.13400

**Published:** 2022-03-30

**Authors:** Rosario Francisco‐Velilla, Azman Embarc‐Buh, Salvador Abellan, Encarnacion Martinez‐Salas

**Affiliations:** ^1^ Centro de Biología Molecular Severo Ochoa CSIC‐UAM Madrid Spain

**Keywords:** cap‐independent translation, host translation shutdown, IRES element, RNA structure, RNA virus, RNA‐binding protein

## Abstract

The genome of viruses classified as picornaviruses consists of a single monocistronic positive strand RNA. The coding capacity of these RNA viruses is rather limited, and thus, they rely on the cellular machinery for their viral replication cycle. Upon the entry of the virus into susceptible cells, the viral RNA initially competes with cellular mRNAs for access to the protein synthesis machinery. Not surprisingly, picornaviruses have evolved specialized strategies that successfully allow the expression of viral gene products, which we outline in this review. The main feature of all picornavirus genomes is the presence of a heavily structured RNA element on the 5´UTR, referred to as an internal ribosome entry site (IRES) element, which directs viral protein synthesis as well and, consequently, triggers the subsequent steps required for viral replication. Here, we will summarize recent studies showing that picornavirus IRES elements consist of a modular structure, providing sites of interaction for ribosome subunits, eIFs, and a selective group of RNA‐binding proteins.

## Graphical abstract

Upon the entry of the viral RNA into susceptible cells, *cis‐* and *trans*‐regulatory elements coordinately determine picornavirus protein synthesis. Early in the infection, the cap‐dependent translation of host mRNAs is shut down, while viral RNA takes advantage of the IRES element, resulting in efficient synthesis of viral proteins. In this review, we discuss RNA structural motifs and host factors influencing viral RNA translation.

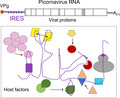

**Abbreviations**
A(n): poly‐A tract
C(n): poly‐C tract
CL: clover leaf
Cre: cis‐replicative element
eIFs: eukaryotic initiation factors
EMCV: encephalomyocarditis virus
EV71: enterovirus 71
FMDV: foot‐and‐mouth disease virus
HAV: hepatitis A virus
HCV: hepatitis C virus
IGR: intergenic region
IRES: internal ribosome entry site
ITAFs: IRES trans‐acting factors
Nt: nucleotide
Pks: pseudoknots
PRF: programmed −1 frameshifting
PTV: porcine teschovirus
PV: poliovirus
RBPs: RNA‐binding proteins
S: small fragment of aphthovirus RNA
SHAPE: selective 2' ‐hydroxyl acylation analyzed by primer extension
uORF: upstream ORF
VPg: viral protein genome

## Overview of the picornavirus genome

Picornaviruses are small, icosahedral, non‐enveloped RNA viruses, which belong to the vast group of positive‐strand RNA viruses. The *Picornaviridae* family includes a number of prominent human and animal pathogens, currently classified into 68 genera and 158 species [1]. Representative genera of this family comprise Enterovirus, Rhinovirus, Hepatovirus, Parechovirus, Kobuvirus, Erbovirus, Cardiovirus, Aphthovirus, and Teschovirus. Other genera of this growing family are Ampivirus, Aquamavirus, Avihepatovirus, Dicipivirus, Megrivirus, Senecavirus, Sapelovirus, and Tremovirus [2]. The Enterovirus genus gathers well‐known pathogens like Poliovirus (PV), human Rhinovirus (HRV), Coxsackie virus 3 (CBV3), and human Enterovirus (EV71). Representative members of the Cardiovirus and Aphthovirus genera are encephalomyocaditis virus (EMCV) and foot‐and‐mouth disease virus (FMDV), respectively. The Teschovirus genus is represented by porcine Teschovirus (PTV).

**Fig. 1:**
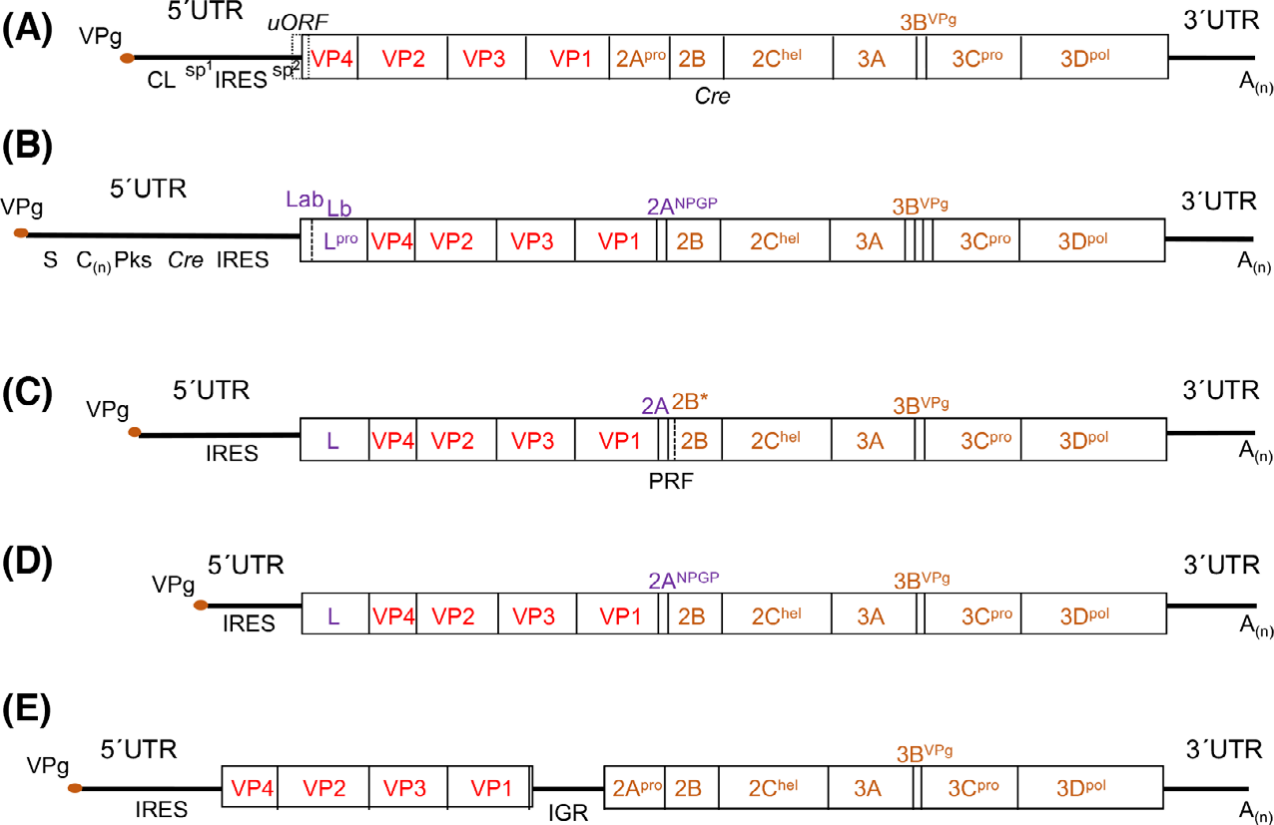
Genome organization of representative members of picornaviruses. Schematic representation of the enterovirus (A), the aphtovirus (B), the cardiovirus (C), the teschovirus (D), and the dicipivirus (E) genome. The thick black line represents the viral RNA (not drawn to scale). Functional and structural RNA elements located at the 5´UTR and 3´UTR are indicated (CL, clover‐leaf; uORF, upstream open reading frame; S, small fragment of aphthovirus; C(n), poly‐C tract; Pks pseudoknots; cre, cis replicative element; IRES, internal ribosome entry site; PRF, programmed −1 frameshifting, IGR, Intergenic region, A(n), poly‐A tract). The picornavirus IRES element varies from 200 to 500 nt long and folds into different secondary structures corresponding to five different types. In enterovirus, two small spacers (sp1 and sp2) separate the CL and the IRES, and the 3´end of the IRES form the functional AUG codon, respectively. The *cre* element of distinct picornaviruses can be found in different positions of the genomic RNA. The empty box depicts the open reading frame encoding a polyprotein. Primary and secondary cleavages of the polyprotein by viral proteases (L^pro^, 2A^pro^ and 3C^pro^) results in the indicated mature viral proteins. Superscripts denote the known function of the protein (VPg, genome‐linked viral protein; pro, protease; pol, polymerase; hel, helicase; NPGP, cis‐active translation termination reinitiation site). Red letters denote the structural proteins (capsid proteins). Orange letters denote conserved function among the non‐structural proteins of the different genera, while violet letters represent specific functions identified in certain picornavirus genus.

The genome of all members of the *Picornaviridae* family consists of an infectious monocistronic single stranded positive RNA [3], with the exception of the Cadicivirus A (genus Dicipivirus), which possesses a dicistronic genome [4]. The picornavirus genome is characterized by the presence of a long uncapped heavily structured 5´UTR, which contains a covalently linked viral protein genome (VPg) at the 5´end (Fig. 1). Common to all picornavirus, the RNA genome harbors an internal ribosome entry site (IRES) element within the 5´UTR responsible for viral protein synthesis [5, 6], and a poly(A) tail at the 3´end [7]. The 3´UTR folds into a stem‐loop structure involved in viral RNA translation and replication [8].

The picornavirus genome encodes a polyprotein, which is proteolytically processed by viral‐encoded proteases through a cascade of primary and secondary cleavages of the polyprotein precursors into 11‐15 mature proteins, depending on the genus [9]. The structural proteins (VP4, VP2, VP3, and VP1) form part of the viral capsid, and the non‐structural proteins mediate polyprotein processing, viral RNA replication, and virus spread. Despite sharing these main features, distinct picornavirus species are characterized by differences within the RNA regulatory elements present in the 5´UTR and the 3´UTR, as well as by differences in the mature viral proteins (see below).

The genomic RNA of Enterovirus (about 8000 nt) is characterized by the presence of a so‐called clover leaf (CL) structural motif (about 90 nt) at the most 5´end of the 5´UTR. The CL is involved in viral RNA replication [10] assisted by the *cis*‐replicative element (*cre*), a stem‐loop of about 20 nt placed within the coding region [11]. A short spacer, enriched in cytosine residues, separates the CL from the IRES element (460 nt). The IRES element directs the translation of the ORF from a functional AUG codon, generating a long polyprotein (about 2330 amino acids), which is cotranslationally and post‐translationally processed into the mature viral proteins. Another poorly conserved spacer is located between the IRES element and the functional AUG codon [12]. Viral‐encoded proteases are responsible for the sequential processing of the polyprotein, ultimately rendering the structural viral proteins (VP4, VP2, VP3, and VP1), and the nonstructural proteins (2A^pro^, 2B, 2C^hel^, 3A, 3B^VPg^, 3C^pro^, and 3D^pol^) (Fig. 1A).

Similar to most Picornaviruses, each Aphthovirus particle consists of a protein capsid comprised of 60 copies of the structural proteins VP4, VP2, VP3, and VP1 surrounding the genomic RNA [13]. The genome (about 8500 nt) contains an extraordinarily long 5'‐UTR, about 1300 nt, which is comprised of RNA elements performing critical functions for the viral replication cycle. From the 5´ to 3´direction, the 5´UTR of FMDV harbors the S fragment involved in RNA replication, a long poly(C) tract, 2‐4 pseudoknots (Pks), the *cre* element, and the IRES element (Fig. 1B). Both the poly(C) tract and the Pks are important for viral infectivity through poorly characterized mechanisms. As it occurs in all Picornaviruses, the viral RNA is synthesized by the virus‐encoded RNA‐dependent RNA polymerase (3D^pol^) assisted by other viral proteins [14].

A distinct feature of FMDV RNA is the presence of two in‐frame evolutionary conserved initiation codons, AUG1 and AUG2, which can be used as initiation sites for protein synthesis, generating the leader (L) proteins, termed Lab and Lb, respectively [15]. Lb protein is made in excess over Lab in infected cells. Remarkably, AUG2 is placed in a more favorable context for protein synthesis. However, improving the Lab context increased the efficiency of translation initiation at the first functional AUG codon [16]. Differences in 48S initiation complex assembly at AUG1 or AUG2 using RNA reporters with the IRES region extended to AUG2, suggested the existence of distinct initiation mechanisms operating at each of these codons [17]. A few additional distinctive features are found in FMDV. First, the Lab and Lb proteins are papain‐like cysteine proteases, which catalyze its hydrolysis from the rest of the polyprotein [18] and also induce the cleavage of the translation initiation factor eIF4G early during infection [19]. Second, the 2A sequence in FMDV is a short peptide of 18 amino acids, which prevents the formation of the peptide bond at the 2A/2B junction [20], using a conserved peptide sequence (NPGP) via a stop‐go translation mechanism. Third, a unique feature of FMDV is the expression of 3 forms of VPg (VPg1, VPg2, and VPg3) [21]; all three are required for enhanced virulence. Nonetheless, in common with all picornavirus, FMDV 2C protein has ATPase activity, and the processing of the viral polyprotein is mostly achieved by the 3C protease (3C^pro^) [22].

Translation of the Cardiovirus genome renders a polyprotein that is cleaved by 3C^pro^, generating the mature viral proteins (Fig. 1C). Cardioviruses also express the L protein, but it does not have protease activity. In some members of this genus, another form of the proteins L^∗^ and 2B^∗^ is expressed from a different open reading frame. The protein 2B^∗^ results from a frameshift activated by the EMCV 2A protein, which selectively binds to a pseudoknot‐like conformation of a programmed −1 ribosomal frameshifting (PRF) [23], and as a consequence, regulates the ratio of structural and non‐structural proteins during cardiovirus infection. On the other hand, the expression of L^∗^ from the Theiler´s murine encephalomyelitis virus (TMEV) is important for viral disruption of interferon signaling (reviewed in [24]).

The genome organization of Teschovirus genus (Fig. 1D) differs from both Enterovirus and Aphthovirus. The 5´UTR of all species within this genus is generally shorter, and the IRES element is placed closer to the 5´end. The first gene product is the L protein, but unlike the Aphthovirus L gene product, it does not possess proteolytic activity. However, similar to FMDV, the Teschovirus genome encodes a 2A^NPGP^ peptide, whereas similar to most Picornaviruses, it only encodes one 3B^VPg^ protein.

In contrast to most Picornaviruses, the genome of members of the Dicipivirus genus is characterized by the presence of an intergenic region (IGR) separating the coding region of structural and nonstructural proteins [4] (Fig. 1E).

In all Picornavirus, the 3A protein delivers the 3B^VPg^ polypeptide to the RNA replication sites. Viral RNA replication requires the covalent attachment to VPg of 5´‐terminal UMP (uracyl monophosphate) to the hydroxyl group of tyrosine, thereby yielding uridylylated VPg (VPgpU and VPgpUpU), an essential and differential step for Picornavirus RNA synthesis, which uses the first 2 adenines of the AAACA sequence of the *cre* element as a template [11, 25]. The 3A protein is anchored to membranes, facilitating viral RNA replication in membrane vesicles. The expression of 3A protein alone disrupts the Golgi apparatus. Earlier studies have shown that Picornaviruses that disrupt the Golgi function, as exemplified by enterovirus [26], are sensitive to Brefeldin A, an inhibitor of vesicle transport between the Golgi and the endoplasmic reticulum (ER). However, both EMCV and FMDV are insensitive to Brefeldin A, and instead, this treatment enhances virus infection [27]. In this respect, a recent study reported that the intracellular membrane trafficking Ras‐related protein Rab1b, a Golgi brefeldin A resistant guanine nucleotide exchange factor 1 (GBF1) effector molecule, and the small GTPase ADP‐ribosylation factor Arf5 proteins contribute to the localization of RNAs containing the FMDV IRES at the ER–Golgi [28], consistent with earlier studies showing the ER localization of RNAs carrying the EMCV IRES element [29].

The 3C protease of all Picornaviruses is a member of chymotrypsin‐like serine proteases, which are characterized by a catalytic triad that comprises a histidine, an acidic residue, and a cysteine. The 3C^pro^ cleavage sites were identified by the sequence alignments of the identified processing sites on the Picornavirus polyprotein. In Enteroviruses, the cleavage site is a dipeptide (Q/G) [30], whereas FMDV 3C^pro^ is more permissive and can accept E/G, E/S, Q/L, Q/I, and Q/T dipeptides (reviewed in [31]). The 3C protease of FMDV and hepatitis A virus (HAV) is responsible for most of the cleavages within the polyprotein coding sequence, but in contrast to the PV 3C^pro^ [30], it does not require 3D^pol^ sequences for any of its processing activities.

The picornavirus 3D protein is the RNA‐dependent RNA polymerase (3D^pol^). Similar to other RNA polymerases, its three‐dimensional structure folds into fingers, palm, and thumb subdomains, with the presence of an NH_2_‐terminal segment surrounding the active site [14]. A detailed structural analysis of the 3D^pol^ protein in the complex with the VPg‐UMP substrate has shown that two magnesium cations together with the catalytic aspartic acid residue participate in the uridylylation reaction. 3D^pol^ catalyzes the initiation of picornavirus RNA replication by the addition of two UMP residues to the Tyr‐3 residue of VPg [32, 33].

The 3'UTR of picornavirus genome is a short region generally consisting of two components: a poorly conserved region of about 100 nt and the poly(A) tail. In FMDV, the deletion of the unique heterogeneous sequence that folds into two stem‐loops blocks infectivity [34]. In contrast, it was reported that the 3'UTR sequence of PV RNA could be deleted, though this deletion severely reduced the RNA replication capacity [35]. Studies on the 3'UTR of cardiovirus RNA have shown evidence for three stem‐loop structures, one of which is essential for virus viability [36].

## Picornavirus translation strategies for evading shutdown of global protein synthesis

Picornaviruses are obligate intracellular parasites that rely on the host translation machinery to produce the proteins required for their replication and spread. Picornavirus viral particles, however, do not contain nonstructural proteins required for viral multiplication. Hence, the first phase of the picornavirus infection cycle requires the translation of the viral RNA, which is governed by the IRES element. This property is shared with other RNA viruses, such as hepatitis C (HCV), a member of the *Flaviviridae* family, as well as members of the families *Dicistroviridae* (CrPV) and *Retroviridae* (HIV‐1), reinforcing the fact that the use of IRES elements represents an optimized strategy for achieving successful viral infection [37, 38]. A key feature of viral IRES elements is that they are active outside of its natural RNA background, promoting cap‐independent activity in different genetic contexts. Indeed, this property was exploited very soon after their discovery to express proteins from artificial bicistronic constructs in different settings [39].

**Fig. 2:**
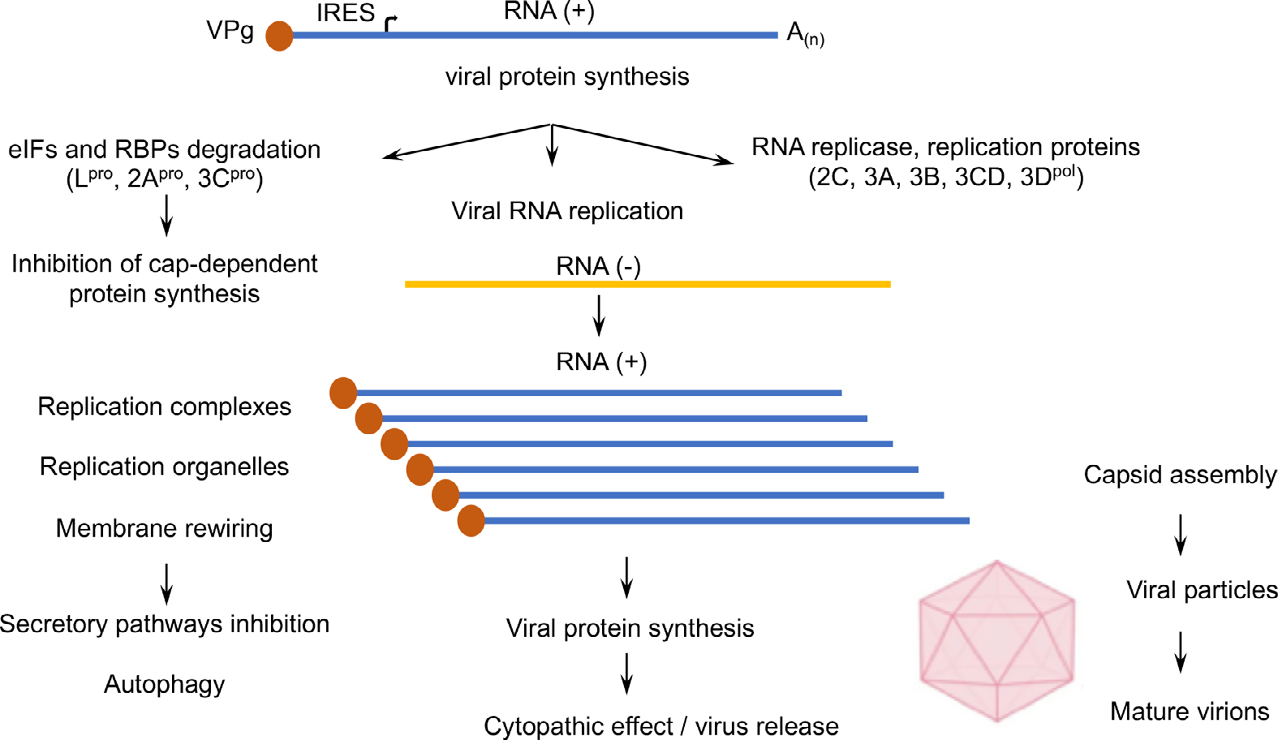
Overview of the picornavirus replication cycle. The first intracellular phase of the picornavirus infection cycle is translation of the viral RNA, governed by the IRES element. This crucial step generates the viral proteins required for the synthesis of the negative strand, RNA (‐), the inhibition of cap‐dependent protein synthesis, the formation of RNA replication complexes and membrane rewiring events, which then lead to modification of cellular activities (inhibition of secretory pathways, autophagy). This is followed by new rounds of positive strand RNA (+) genome and viral protein synthesis. Assembly of the structural proteins into viral capsids precedes packaging of the viral RNA genome into mature virions. For simplicity, only the main steps have been represented.

Similar to many other RNA viruses, picornaviruses co‐opt multiple host RNA‐binding proteins (RBPs) that assist with the recruitment of viral RNAs for viral protein synthesis and also with the assembly of the complexes regulating viral RNA synthesis [37]. The picornavirus genome encodes a long polyprotein, which is rapidly processed by virus‐encoded proteases into structural and nonstructural proteins through a cascade of primary and secondary cleavages of the polyprotein precursors (reviewed in [9, 31]). This initial step produces the viral proteins required for triggering a cascade of events required for the viral replication cycle, which includes the synthesis of the negative strand of the viral RNA, the inhibition of cap‐dependent protein synthesis, the formation of RNA replication complexes, and the modification of cellular activities that include membrane rewiring and the inhibition of the secretory pathway (Fig. 2). Collectively, these processes entail the generation of new rounds of positive strand RNA genomes and the formation of virus particles. Following the assembly of the structural proteins into viral capsids, the viral RNA genome is packaged into mature virions by still poorly characterized mechanisms.

In marked difference with the vast majority of cellular mRNAs, all picornaviruses contain regulatory elements in their genome that allow the recruitment of the translation machinery to the appropriate translation initiation codon using a cap‐independent mechanism, governed by the IRES element [40]. IRES elements are highly structured RNA regions that recruit the translation machinery internally in a process guided by RNA structural motifs, a subset of eIFs, and cellular RBPs acting as IRES trans‐acting factors (ITAFs). However, IRES elements differ in primary sequence, RNA structure, and protein factor requirements. The ribosome recruitment of representative members of the picornavirus IRES elements, such as enterovirus and cardiovirus, requires the translation initiation factors eIF2, eIF3, eIF4A, eIF4G, eIF4B, and eIF1A, but not eIF4E. Thus, the IRES‐dependent translation initiation mechanism differs from the cap‐dependent mechanism operating in most cellular mRNAs [41].

The cap structure present in the vast majority of cellular mRNAs allows the binding of the eIF4F trimeric complex (the cap‐binding protein eIF4E, the RNA helicase eIF4A, and the scaffolding protein eIF4G). The recognition of the 5´cap by eIF4E allows the recruitment of the 43S complex (consisting of the initiator methionine tRNA (Met‐tRNA_i_), eIF2, and GTP) and the small 40S ribosomal subunit, assisted by additional eIFs. The interaction of eIF4G with the poly(A)‐binding protein (PABP), in turn bound to the poly(A)‐tail of mRNAs, circularizes the 3´ and 5´ ends of the mRNA, stimulating translation. Under normal conditions, the 43S complex scans the 5´UTR of the mRNA until an initiator start codon (AUG) is located in optimum sequence context. AUG recognition triggers the joining of the 60S ribosomal subunit and eIF release, leading to the assembly of a translation elongation competent 80S ribosome [41].

IRES‐mediated translation initiation is an important part of the strategy used by picornaviruses to recruit the translation machinery when cap‐dependent translation is inhibited by the action of viral proteases that induce the cleavage of eIFs and RBPs [37], and also by the cellular antiviral response [42]. Picornavirus infection induces the shutdown of host gene expression through the action of viral proteases that cleave factors required for cap‐dependent translation initiation, such as eIF4G, PABP, and eIF5B (reviewed in [42, 43]). The cleavage of the eIF4G factor by the FMDV L^pro^ and rhinovirus 2A^pro^ results in the separation of the N‐terminus of the eIF4G protein carrying the binding sites for PABP and eIF4E, hence preventing cap‐dependent translation [44, 45]. On the contrary, the C‐terminal region of eIF4G carrying the binding sites for eIF4A and mitogen activated protein *kinase‐*interacting *kinase 1* (mnk1) directs IRES‐driven protein synthesis [46, 47]. However, in marked difference with other picornaviruses, EMCV does not induce the cleavage of eIF4G. Instead, the inhibition of cap‐dependent translation is caused by 4E‐binding protein (4E‐BP) dephosphorylation [48], hampering the interaction of eIF4G with eIF4E [49].

In addition to eIFs, various ITAFs are targets of picornavirus proteases, such that the resulting cleavage products perform functions different from the uncleaved protein [42]. In other cases, post‐translational modification of the proteins or changes in the associated factors in response to stress play a role in modulating IRES activity, either directly or indirectly [38, 43].

## Host factors involved in picornavirus RNA translation

The replication and translation of the picornavirus genome takes place in the cytoplasm. Beyond the C‐terminal moiety of eIF4G, picornaviruses subvert a wide variety of nuclear and cytoplasmic RBPs to assist viral RNA translation using diverse strategies [42, 43]. These strategies are in part facilitated by the delocalization of RBPs to the cytoplasm in infected cells, an event that increases the pool of functionally active factors for viral RNA translation. Soon after the discovery of picornavirus IRES elements in 1988 [5, 6], it was found that full IRES activity required the action of host factors, as exemplified by the polypyrimidine tract‐binding protein (PTB), poly(rC)‐binding protein 2 (PCBP2), ErbB3‐binding protein 1 (Ebp1, also known as ITAF_45_ and PA2G4), or the cold‐shock domain containing E1 (CSDE1, also known as Unr), among others [50, 51, 52, 53, 54], reviewed in [38, 55].

**Fig. 3:**
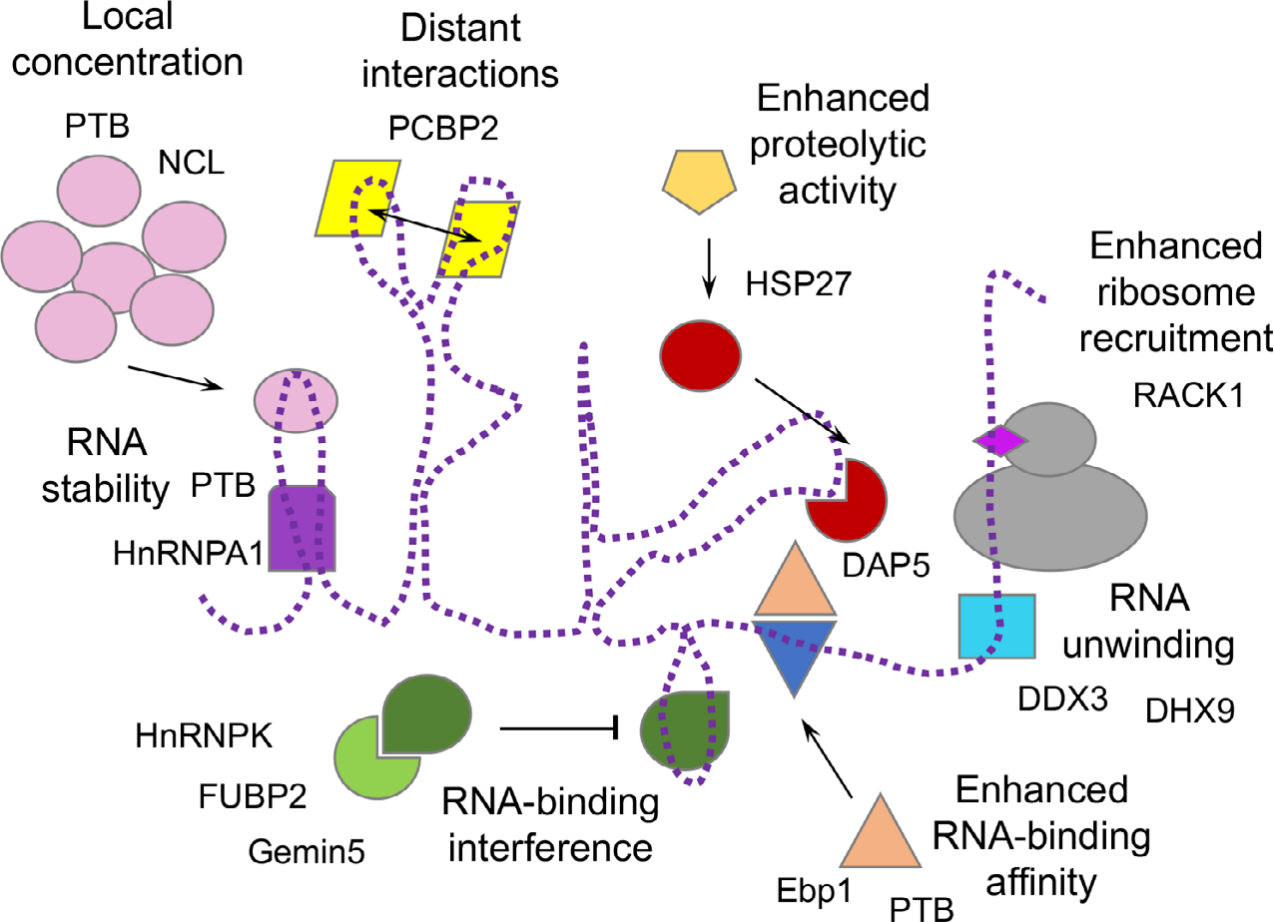
Different functions proposed for RNA‐binding proteins interacting with picornavirus IRES elements. Proteins acting as repressors or stimulators of IRES activity have been represented with different colors according to its activity (changing local protein concentration, facilitating RNA stability, promoting RNA unwinding, stabilizing distant interactions, increasing RNA‐binding affinity by an auxiliary factor, interfering binding by protein‐protein interaction, or enhancing ribosome recruitment). Examples of the proteins referred to in the text are shown in the figure.

Proteins recycled for picornavirus IRES activity in infected cells can act either as stimulators or repressors of viral RNA translation. The enhancer or repressor activity of the auxiliary host factors depends upon the mechanism of action supporting RNA‐protein or protein‐protein interactions. Indeed, different proteins can modulate IRES activity through changes in the local concentration of the protein, promoting unwinding of the IRES secondary structure, remodeling the RNA structure by stabilizing distant interactions between structural motifs and maintaining the active RNA conformation, inducing the proteolytic activity of viral proteases required to cleave repressor proteins or eIFs, enhancing RNA binding affinity, sequestering negative factors, and facilitating ribosome recruitment, among other possibilities (Fig. 3). A few examples of recently reported proteins involved in these diverse strategies are discussed below.

Similar to other positive strand RNA viruses, picornaviruses have evolved strategies to target nucleo‐cytoplasmic trafficking of host proteins and RNA, and as a result, to increase the availability of factors required for their multiplication cycle. Among well‐known targets of picornavirus are the nucleoporins, the critical components of the nuclear pore. The disruption of the nuclear pore during early infection impairs active nucleo‐cytoplasmic transport in both directions [56]. Nuclear pore targeting leads to nuclear translocation of cytoplasmic proteins that induce innate immune responses and retain host mRNA in the nucleus, thereby preventing host responses. The host factors required for viral IRES‐mediated translation can be limiting during the initial stages of viral infection until the virus‐encoded protease activity inactivates cap‐dependent translation. In addition, limited amounts of ITAFs may be available in the cytoplasm until the nuclear membrane is disrupted, such that the initial round of viral RNA translation can be facilitated by alternative cytoplasmic factors. In the initial stages of CVB3 infection, it has been proposed that a basal level of translation is carried out by the cytoplasmic protein, death‐associated protein 5 (DAP5) [57], followed by the C‐terminal eIF4GI in the subsequent stages. Of note, DAP5 is cleaved by the viral 2A^pro^ into N‐ and C‐terminal parts, so that the N‐terminal fragment retains the middle domain of eIF4G. However, since DAP5 has a lower affinity for the CVB3 IRES compared with authentic C‐terminal eIF4GI, the recruitment of other factors such as eIF4A is required for the full IRES function.

Studies carried out over the years have shown that picornavirus infection induces stress response, consequently leading to overall gene expression regulation (reviewed in [37, 55]). Nucleolin (NCL) is one of the stress‐responsive RBPs that delocalizes to the cytoplasm in infected cells. This protein interacts with the FMDV IRES, promoting the initiation of translation [58]. Interestingly, NCL also modulates IRES‐dependent translation of Seneca Valley virus (SVV) and classical swine fever virus (CSFV), but not cellular or vesicular stomatitis virus (VSV) cap‐dependent translation, suggesting a general activity on viral IRES elements.

## Regulation of picornavirus translation by host factor proteolysis

There are various examples of RBPs interacting with picornavirus IRES elements, which are proteolyzed during infection, remodeling their activity on translation after cleavage. Among others, known examples include the PTB protein, the far‐upstream element binding protein 2 (FUBP2), FUBP1, the heterogeneous nuclear ribonucleoprotein (hnRNPK), PCBP2, the Ras GTPase SH3 domain binding protein 1 (G3BP1), and Gemin5 (reviewed in [55]). In the case of PTB, the PV 3C protease recognizes the three isoforms of this protein, generating truncated polypeptides that repress PV IRES activity [59]. Similar results have been reported for PCBP2 [60]. The opposite effect is observed in FUBP2, where the cleavage of this repressor protein in EV71‐infected cells results in a fragment that loses its C‐terminal region, behaving as an IRES stimulator [61]. Likewise, Gemin5 is cleaved in FMDV‐infected cells by the action of the leader protease, rendering two detectable C‐terminal products, p85 and p57, from sequential cleavage events [62]. In this case, while the full‐length protein behaves as a negative regulator of both FMDV and hepatitis C virus (HCV) IRES‐dependent translation, the p85 fragment upregulates the IRES activity [63]. This scenario is reminiscent of eIF4GI proteolysis in PV‐infected cells, where the cleavage of the full‐length protein abrogates cap‐dependent translation but stimulates IRES‐dependent activity [64].

Instances of host factors promoting the activity of viral proteases on eIF4G have been recently reported, as shown by the heat shock proteins, HsP27 and Hsc70. Hsp27 is upregulated in response to EV71 infection [65]. This protein appears to regulate IRES activity by two different mechanisms. First, it promotes 2A^pro^‐mediated eIF4G cleavage, favoring cap‐independent translation of the viral RNA. Second, the knockout of Hsp27 blocks hnRNP A1 translocation from the nucleus to the cytoplasm, abolishing the interaction of hnRNP A1 with the IRES element. Similarly, Hsc70 interacts with 2A protease to promote eIF4G cleavage, likely activating EV71 IRES activity [66].

Another strategy facilitating picornavirus RNA translation is exemplified by G3BP1 and hnRNP K proteins, which negatively regulate FMDV translation [67, 68]. hnRNP K binds directly to the FMDV IRES element through the KH2 and KH3 domains, interfering with the recognition of PTB, thereby inhibiting IRES‐mediated translation. However, hnRNP K‐mediated inhibition is antagonized by 3C^pro^ cleavage in infected cells, such that the N‐terminal cleavage product retains inhibitory effects on IRES activity, whereas the C‐terminal cleavage product becomes a positive regulator of FMDV replication. The cleavage of G3BP1 by FMDV 3C protease renders two fragments that inhibit cap‐ and IRES‐dependent translation, but the Ct‐G3BP1 fragment shows a stronger effect on IRES‐dependent translation. The assembly of complexes with G3BP1 results in a significantly reduced local flexibility of the IRES element, consistent with the negative effect of this protein [68].

## Indirect modulation of picornavirus translation

In addition to the strategies mentioned above, it has been reported that PV exploits specific ribosomal proteins, such as the ribosomal protein receptor for activated C kinase 1 (RACK1), to enhance ribosome recruitment, and therefore favor the translation of the viral proteins in infected cells [69]. Another example of a host factor stimulating IRES activity is the ribosomal protein L13 (RPL13). RPL13 modulates translation of several IRES elements, namely, FMDV, SVV, and CSFV. This interaction is regulated by the DEAD box helicase DDX3, which contributes to the recruitment of eIF3e and eIF3j to the IRES element and also cooperates with RPL13 to support the assembly of 80S ribosomes for the translation initiation of viral mRNA [70]. In particular, the depletion of DDX3 disrupts the binding of RPL13 to the FMDV IRES element, whereas the reduction in RPL13 expression impairs the ability of DDX3 to promote IRES‐driven translation.

The permissive RNA‐binding capacity of ITAFs in conjunction with the multitasking activity of these factors, allows interaction with multiple targets, either RNA or proteins. The different steps of the picornavirus multiplication cycle are closely interconnected. Accordingly, there are examples of host factors interacting with IRES elements, which affect viral RNA replication, rather than RNA translation. For instance, the heterogeneous nuclear ribonucleoprotein L (hnRNP L) interacts with the FMDV IRES to inhibit viral RNA replication [71]. Given that hnRNP L co‐immunoprecipitates with RNA‐dependent RNA polymerase (3D^pol^) in infected cells, it has been suggested that the inhibitory effect of hnRNP L on FMDV growth is presumably exerted through viral RNA synthesis.

## Additional strategies of translation control exploited by picornaviruses

The 5´UTR of the picornavirus genome is characterized by the presence of many unused AUG codons, as shown in early studies of poliovirus RNA [72]. In particular, enterovirus genomes harbor a conserved upstream ORF (uORF) within the spacer that separates the IRES element from the functional start codon (Fig. 1A), just upstream of the main ORF encoding the polyprotein. Although the enteroviral proteins were thought to derive exclusively from the proteolytic processing of the polyprotein, recent ribosome profiling data indicated low level expression of a small uORF protein in cells infected with echovirus 7, PV, or EV [73]. Furthermore, echovirus mutants defective in the expression of this short protein are attenuated compared to the wild‐type at late stages of infection in differentiated human intestinal organoids, where the membrane‐associated uORF protein facilitates virus release. Although non‐enveloped viruses are thought to exit cells via cell lysis using the conventional secretory pathway for the delivery of cytoplasmic molecules, it has been shown that enteroviruses subvert the host autophagy pathway [74], resulting in intracellular membrane vesicles containing virus that can be released from cells [75]. Hence, the virus titer decrease observed at late stages of infection, together with the increased proportion of membrane‐associated virus in the absence of the small protein suggests that this small peptide may serve as a membrane disruptor that allows virus particle release from vesicles in gut epithelial cells. However, it remains elusive as to whether this situation applies to other picornaviruses, which also infect the gut.

Another strategy proposed to modulate picornavirus translation relies on slowing down the translation kinetics of the viral RNA. The rationale behind this possibility relies on the observation that the rate of translation elongation can regulate cotranslational protein folding [76]. Translation kinetics depends on the rate of ribosome traffic on the mRNA, which also depends on tRNA abundance. Usually, the most abundant tRNAs correspond to preferred codons and facilitate translation elongation, whereas less abundant tRNAs correspond to rare codons and slow down translation speed. In contrast with other picornaviruses, HAV codon usage is biased and deoptimized relative to its host [77]. However, the HAV capsid shows enhanced physical stability, presumably related to the slow translation elongation rate imposed by its codon usage. In support of this notion, HAV mutants with changes in capsid codon usage show differences in the local rate of translation, which appears to influence the folding of newly synthesized protein. Additionally, the HAV IRES is weaker than most picornavirus IRES elements, requiring both eIF4G and eIF4E [78, 79]. Therefore, the deoptimized HAV codon usage may be considered a strategy to overcome competition for tRNAs within the cell environment, and perhaps, to remain undiscovered in the infected cell.

## Structural features of viral IRES elements

Studies conducted since its discovery in 1988 have shown that IRES elements are *cis*‐acting RNA regions that promote internal initiation of translation using diverse cap‐independent mechanisms [80, 81, 82]. Most viral IRES elements adopt complex RNA structures, which serve as the anchoring site for the ribosome guided by RNA‐RNA and/or RNA‐protein interactions [40, 83]. In this respect, it is important to note that IRES elements function as an RNA machine (IRESome), recruiting eIFs and ITAFs along its RNA structure. Therefore, short regions/sequences of the element do not exhibit the activity produced by the entire element [84], due to the loss of a correct IRESome assembly.

Despite performing the same function, the IRES elements present in the genome of different RNA virus families lack overall conserved features, and differ in the requirement of factors to assemble 48S initiation complexes [80, 81, 82, 83]. Furthermore, even within the same genus, single nucleotide substitutions appearing in the process of viral RNA replication can alter the folding and/or the RNA‐protein interaction, therefore modulating IRES activity [85, 86]. In addition, it has been proposed that recombination events occurring during picornavirus coinfection in nature generate IRES elements with unique properties that allow novel tissue tropisms and/or host‐range spectrum [87]. The requirement of specific ITAFs for IRES function, and their dissimilar presence among different cell types, might contribute to cell‐type‐dependent IRES function, and also restrict cross‐kingdom IRES activity.

Viral IRES elements have been classified into different types according to their mechanism of initiation. The simplest mechanism occurs by direct interaction of the IRES with the 40S ribosomal subunit [83]. A more complex one depends upon IRES binding to eIFs and RBPs, allowing the recruitment of the 40S subunit [40, 80]. Currently, there are five types of picornavirus IRES elements according to the secondary structure organization and the mechanism of action. The RNA genomes of enterovirus (PV, CVB3, EV71, and human rhinovirus (HRV)) contain type I IRES elements, while type II IRES elements are present in Cardiovirus (EMCV) and Aphthovirus (FMDV) RNAs. Both type I and II IRES are independent of eIF4E but require the C‐terminal region of eIF4G, eIF4A, eIF2, and eIF3 to assemble 48S initiation complexes *in vitro* [46, 88]. However, translation initiation driven by type III, present in HAV RNA, depends on the integrity of eIF4G [79]. In this case, eIF4E binding to eIF4G generates a high‐affinity binding conformation of the eIF4F complex for the HAV IRES element. Additionally, eIF4E‐eIF4G binding stimulates the rate of IRES RNA unwinding by eIF4A [89].

Type IV IRES elements are eIF4G‐independent, but depend on eIF2 and eIF3. In fact, these group of IRES elements were designated HCV‐like because of the close similarity with the HCV IRES [90]. The IRES element classified as type V is present in the Aichivirus (AiV) genomic RNA, which belongs to Kobuvirus genus. This IRES element appears to be a chimera between type I and type II [91]. Its function involves interaction with eIF4G, although the eIF4G‐interacting domain is structurally distinct. Also, AiV IRES activity is enhanced by PTB, similar to Type 1 and Type 2 IRES elements. Unlike all known IRESs, the AiV IRES depends on the helicase DHX29 due to sequestration of its initiation codon in a stable hairpin [91].

## RNA structural elements that impact on protein binding

**Fig. 4:**
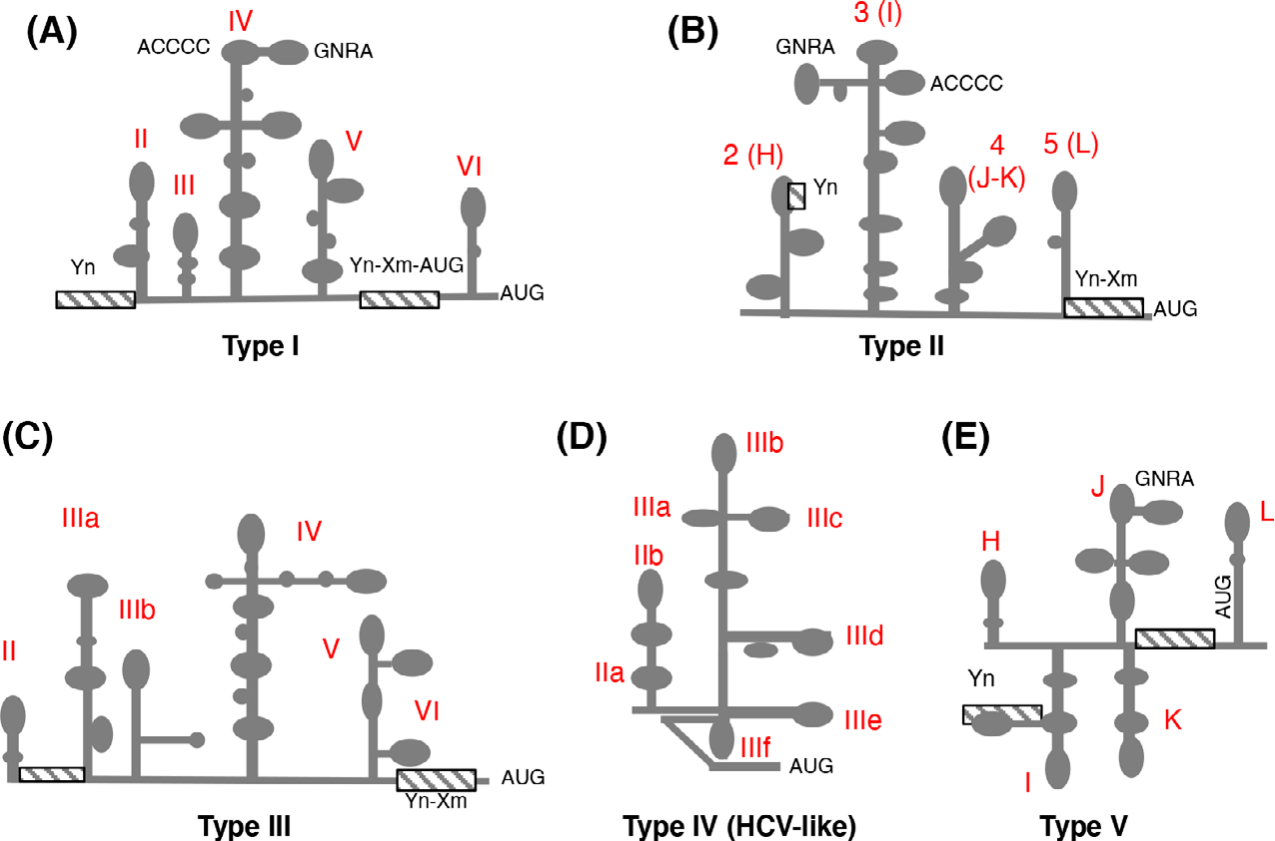
Secondary structure of picornavirus IRES elements. Type I is typically found in enterovirus (A), type II is found in aphthovirus and cardiovirus (B), type III is only present in Hepatitis A virus (C), type IV, also called HCV‐like, is found in teschovirus (D), and type V is found in Aichivirus (E). The locations of domains, sub‐domains and RNA motifs of the five type of picornavirus IRES elements referred to in the text are indicated.

The secondary structure of picornavirus IRES elements has been extensively analyzed over the years using different approaches [78, 91, 92, 93, 94, 95]. Briefly, the enterovirus IRES elements are organized in domains designated II to VI, which adopt stem‐loop structures generally including internal bulges [95, 96] (Fig. 4A). Domain II folds as a stem‐loop that harbors a conserved internal bulge (AUAGC motif) [97]. Domains III and VI are rather variable compared to domains IV and V. Notably, domain IV (about 200 nt) adopts a cruciform structure that includes two essential motifs at its apical subdomain, an internal C‐rich loop and a GNRA (N stands for any nucleotide, and R for purine) tetraloop [98, 99]. Domain V (110 nt) consists of a hairpin with an internal loop and provides the binding site for two critical proteins, eIF4G and PTB [88, 100]. Toward the 3´end of the IRES, domain VI comprises a stem‐loop containing a conserved AUG codon (586AUG in PV) in a poor initiation context. This AUG plays an important role in stimulating attachment of 43S ribosomal preinitiation complexes to the viral mRNA, which then scan the 5´UTR toward the polyprotein initiation site downstream (743AUG in PV). A spacer of 18‐20 nt separates a nonfunctional AUG triplet from the conserved pyrimidine tract, about 30 nt to 160 nt upstream of the initiation codon. In PV and other enteroviruses, the AUG placed in domain VI is followed by a 65‐codon uORF that overlaps the polyprotein ORF by 38 nt [100].

Previous studies reported that the AUG placed in domain VI was not utilized for initiation, and that the 6.5–9.0 kDa protein resulting from uORF translation was not detected in infected cells. However, recent data using selective 2'‐hydroxyl acylation analyzed by primer extension (SHAPE) [95] and base‐specific chemical probes revealed novel structural features on the enterovirus IRES element. These new properties include realignment of major domains, long‐range interactions between domains II and III presumably involving high order structural elements. In addition, this study unveiled the presence of an intrinsically disordered RNA region (IDRR), 20 nt long, within domain V encompassing the conserved pyrimidine‐rich region downstream of domain V with the motif Yn‐Xm‐AUG (where Yn is 8 to 10 pyrimidines, Xm is 18 to 20 nucleotides, and AUG is a cryptic start codon) [95]. These structural features suggest a hierarchical folding process that links structure to function directed by genomic RNA during enterovirus infection.

The relevance of the enterovirus IRES function for viral infection was highlighted earlier by the discovery of specific mutations within domain V of the PV IRES that inhibited viral RNA translation and also induced poliovirus attenuation properties in Sabin vaccine strains [101]. As mentioned earlier, domain V contains binding sites for two proteins needed for IRES function, eIF4G and PTB [100]. Recent studies have shown that the Sabin mutants reduce the equilibrium dissociation constants of eIF4G and PTB to the PV IRES element by up to 6‐fold, such that the apparent affinity of an active eIF4G/eIF4A/eIF4B helicase complex for the IRES element is severely reduced [101]. However, another Sabin mutant did not alter the rate of eIF4A‐dependent helicase activity, suggesting that the latter mutant primarily reduces the affinity, rather than activity, of the unwinding complex [102]. Moreover, eIF4G overexpression in susceptible cells overcomes the attenuation of a replicon harboring the IRES mutant, providing a framework for understanding the mechanism of PV attenuation.

The RNA structure of type II IRES elements is also arranged in modular domains, designated 2 to 5 (or H to L, respectively) [103, 104] (Fig. 4B). Domain 2 contains a conserved pyrimidine tract that provides a binding site for the PTB protein [50, 51]. Domain 3 is a self‐folding cruciform structure [105]. The basal region of this domain consists of a long stem interrupted with bulges that includes several non‐canonical base pairs and a helical structure essential for IRES activity. Domain 4 is organized into two hairpin‐loops, which contain the binding site for eIF4G [46, 47], while domain 5 provides the binding site for eIF4B and PTB [106, 107]. It consists of a phylogenetically conserved hairpin followed by a long pyrimidine tract (9‐11 nt) and a variable single‐stranded stretch of nucleotides immediately upstream of the functional AUG codon [104].

**Fig. 5:**
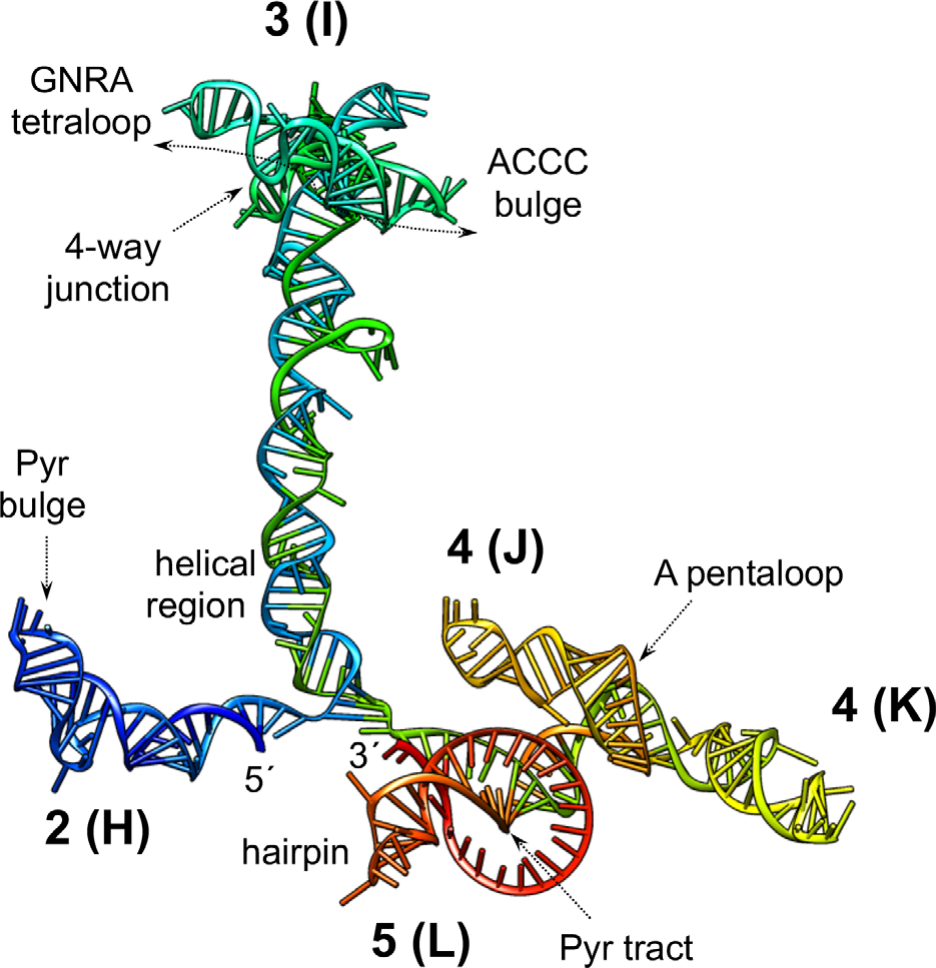
RNA structure model of the FMDV IRES element. Predicted three‐dimensional structure model generated for the IRES, imposing SHAPE reactivity values obtained for the free RNA in solution. Domains 2, 3, 4 and 5, and subdomains J and K of domain 4 are indicated. The 5´ and 3´end of the IRES are indicated. Arrows depict the position of the GNRA tetraloop, ACCC bulge, 4‐way junction, A‐pentaloop, helical region, hairpin and bulges referred to in the main text. Adapted from [130].

Sequence comparison of FMDV field isolates reveals shared conserved motifs exposed on loops and the junctions connecting stems, strongly supporting the hypothesis that the secondary structure is evolutionary constrained to deliver its function [94]. Accordingly, conserved RNA motifs such as the GNRA tetraloop placed in domain 3 (or I) of type II elements determine the IRES architecture [108, 109, 110] (Fig. 5). Furthermore, structural analysis of the J‐K region of the EMCV IRES element in solution revealed that the A‐pentaloop bulge plays a crucial role as a docking site for base‐pair receptors in concerted action of all subdomains [111]. The conformation of the A‐pentaloop resembles the GNRA tetraloop, except that the G is substituted by A‐A dinucleotide. Interestingly, resolution of the crystal structure of the HAV IRES domain V revealed a three‐way junction structure, topologically organized by an adenine‐rich stem‐loop motif [112]. This motif shows a striking similarity to a circularly permuted form of the J‐K domain of EMCV, suggesting a common strategy for organizing the RNA architecture of this domain, also present in the FMDV IRES element. The topological conservation observed among all these IRES elements implicates a three‐way junction as an architectural scaffold to pre‐organize helical domains for recruiting the translation initiation machinery. The structural similarity of the GNRA tetraloop and the A‐pentaloop motifs opens the question of whether they could derive from RNA modules subjected to genetic changes during evolution. It is noteworthy that RNA flexibility is a prominent feature of picornavirus IRES elements [80]. Consistent with this, analysis of the RNA structure in solution using novel dimetallic compounds identified 4‐way and 3‐way junctions within flexible regions of the FMDV and HCV IRES RNA conformation [113].

A wide variety of RNA virus genomes fold into complex structures that include long‐range RNA‐RNA interactions relevant to control critical steps of the viral cycle [114]. In the case of picornaviruses, initiation of translation driven by the IRES element is stimulated by sequences located on the 3' end of the viral RNA [115, 116]. Long‐distant RNA‐RNA interactions were supported by gel mobility‐shift data obtained *in vitro* using specific transcripts [117] and later *in vivo* using SHAPE methodology [118].

The secondary structure of the HAV IRES element (a single member of type III) comprises six domains [78] (Fig. 4C). This IRES element is about 730 nt, significantly longer than type I and II. However, the HAV IRES is weaker than both type I and II, and its activity depends on the cell type [119]. Domains IV and V of the HAV IRES and the pyrimidine‐tract located upstream of the initiation codon form the minimal core of the IRES element. This is in contrast to type II IRES where domain IV and V alone harbor negligible IRES activity [84].

Type IV IRES elements are found in multiple picornavirus genera, including Teschovirus, Sapelovirus, Senecavirus, Tremovirus and Avihepatovirus [82, 84]. This type of IRES element spans around 300 nt, though they are more variable in length than types I and II. Similar to the HCV IRES, type IV IRES elements are organized in domains II, III, and IV [120, 121] (Fig. 4D), and their function is independent of eIF4G. In particular, domain III is a long stem loop that includes several subdomains and a four‐way junction; this domain is responsible for the interaction with eIF3, and the 40S ribosomal subunit (reviewed in [87]). The IRES elements classified as type V are also spread in several picornavirus genera. Curiously, the secondary structure resembles type I and II [91, 122] (Fig. 4E), suggesting that they could have been generated by recombination events.

## Mechanistic attributes of picornavirus IRES elements modulated by RNA‐binding proteins

Host RBPs can modulate IRES function in multiple ways. For instance, they can act as RNA chaperones that promote specific conformations of the IRES structure, stabilize the interaction of the IRES with eIFs, mediate recruitment of the 40S ribosomal subunit, unwind RNA secondary structure near or at the start codon, and/or titrate IRES ligands (Fig. 3).

As already mentioned, the RNA architecture imposed by the presence of specific structural domains mediates IRES function (reviewed in [55]). One of the widely conserved motifs of viral IRES elements is the polypyrimidine tract, which provides the binding site for PTB [51, 91, 123]. Some picornavirus IRES elements classified as types I and II have two polypyrimidine tracts placed at each end of the IRES region (Fig. 4), such that the RNA recognition motifs (RRM) of PTB bind to the IRES element constraining the RNA structure in the orientation required for protein synthesis stimulation [124]. It has been recently suggested that a nucleotide substitution of cytosine for guanine (C/G) within the polypyrimidine tract of the IRES impairing PTB binding endows FMDV with temperature‐sensitive and attenuation phenotypes [125]. Interestingly, natural hosts inoculated with viruses carrying the C/G mutation on the IRES element showed no clinical signs, viremia, virus excretion, or viral transmission, but still elicited a potent neutralizing antibody response that produced complete protection.

Another example of a protein mediating IRES activity is PCBP2, a ubiquitously expressed RBP comprising three ribonucleoprotein K homology (KH) domains. The PCBP2 binding site in the enterovirus IRES elements is the ACCCC loop, a conserved motif located in domain IV in close proximity to the GNRA tetraloop [85, 109]. The PCBP2 protein appears to assist eIFs in reconstitution assays with type I IRES elements by favoring a functional RNA structure conformation [100]. Another piece of evidence for the requirement for PCBP2 is found in the cadicivirus IRES element, that harbors an essential GNRA tetraloop and also requires this factor, suggesting that PCBP2 could act in concerted action with the GNRA tetraloop to enhance internal initiation [126]. Consistent with this idea, structural analysis of a complex containing the full‐length PCBP2 with domain IV revealed a compact globular complex of the protein interacting with the cruciform RNA via KH domains [127]. This three‐dimensional structure indicates that the GNRA tetraloop confers stability to the AUCCC loop to enhance PCBP2 binding. Alternatively, PCBP2 binding could present the GNRA tetraloop in an optimal way for establishing distal interactions (Fig. 3).

A large diversity of RBPs involved in nuclear and cytoplasmic RNA‐dependent processes have been identified associated with viral IRES elements, as exemplified by Ebp1, the far upstream element‐binding protein 1 (FBP1) and FBP2, the heterogeneous nuclear ribonucleoprotein A1 (hnRNPA1), G3BP1, and Unr (reviewed in [38, 55]). However, the number of host factors identified to be involved in viral RNA translation is constantly growing due to the development of new approaches for studying the function of traditional and non‐conventional RNA binding proteins in infected cells [128, 129]. Of note, most of these proteins interact with multiple RNA targets that may respond in a coordinated manner to changes in the cell environment as well as to intracellular signals, such as those caused by picornavirus infections. Hence, understanding how different combinations of these proteins bind to the IRES element to assemble a functional IRESome, and also how changes in status, concentration, and/or post‐translational modification of these factors modulate IRES activity depending on the cell type and the infection phase, will bring new insights into the complex and diverse strategies used by picornaviruses, and other RNA viruses, to express their genome.

## Concluding remarks

As picornaviruses have small genomes, their expression depends on outcompeting host cellular machinery to progress the different steps of the viral replication cycle. These strategies include the cleavage and/or post‐translation modification of eIFs and RBPs resulting in strong shutdown of the general cap‐dependent translation initiation. In contrast to the host mRNA, a key stage of the picornavirus cycle is translation of the incoming genomic RNA governed by IRES elements. Regarding IRES function, it is important to note that the functional features of RNA molecules are established in their three‐dimensional structure, but also in their ability to acquire distinct conformations in response to specific signals [130]. Remarkably, IRES elements harbor conserved RNA motifs essential for viral infection, which provide the binding site for a large number of RBPs, and may constitute specific targets for antiviral compounds [131, 132]. Despite numerous studies, understanding the complexity of RNA‐protein interactions facilitating the expression of viral proteins in infected cells remains elusive. However, some sequences impacting RNA structure and RNA‐protein interactions important for IRES‐driven translation are conserved, raising the question of whether IRES elements could consist of basic building blocks, upon which evolutionary selection acts to generate distinct functional elements with different properties. The recent advances in genomics and proteomics will help in the discovery of new viral genomes containing IRES elements and in characterizing how these elements interact with host factors to achieve full activity.

## Conflict of interest

The authors declare no conflict of interest.

## Author contribution

EMS conceived and wrote the manuscript with comments from all authors (RF‐V, AE‐B, SA).

## Data Availability

Data accessibility is available upon request.
